# Mobile electroencephalography captures differences of walking over even and uneven terrain but not of single and dual-task gait

**DOI:** 10.3389/fspor.2022.945341

**Published:** 2022-10-06

**Authors:** Nadine Svenja Josée Jacobsen, Sarah Blum, Joanna Elizabeth Mary Scanlon, Karsten Witt, Stefan Debener

**Affiliations:** ^1^Neuropsychology Lab, Department of Psychology, School of Medicine and Health Sciences, University of Oldenburg, Oldenburg, Germany; ^2^Hörzentrum Oldenburg GmbH, Oldenburg, Germany; ^3^Cluster of Excellence Hearing4all, Oldenburg, Germany; ^4^Branch for Hearing, Speech and Audio Technology HSA, Fraunhofer Institute for Digital Media Technology IDMT, Oldenburg, Germany; ^5^Department of Neurology and Research Center Neurosensory Science, School of Medicine and Health Sciences, University of Oldenburg, Oldenburg, Germany

**Keywords:** mobile EEG, ERSP, gait, dual-task, terrain

## Abstract

Walking on natural terrain while performing a dual-task, such as typing on a smartphone is a common behavior. Since dual-tasking and terrain change gait characteristics, it is of interest to understand how altered gait is reflected by changes in gait-associated neural signatures. A study was performed with 64-channel electroencephalography (EEG) of healthy volunteers, which was recorded while they walked over uneven and even terrain outdoors with and without performing a concurrent task (self-paced button pressing with both thumbs). Data from *n* = 19 participants (M = 24 years, 13 females) were analyzed regarding gait-phase related power modulations (GPM) and gait performance (stride time and stride time-variability). GPMs changed significantly with terrain, but not with the task. Descriptively, a greater beta power decrease following right-heel strikes was observed on uneven compared to even terrain. No evidence of an interaction was observed. Beta band power reduction following the initial contact of the right foot was more pronounced on uneven than on even terrain. Stride times were longer on uneven compared to even terrain and during dual- compared to single-task gait, but no significant interaction was observed. Stride time variability increased on uneven terrain compared to even terrain but not during single- compared to dual-tasking. The results reflect that as the terrain difficulty increases, the strides become slower and more irregular, whereas a secondary task slows stride duration only. Mobile EEG captures GPM differences linked to terrain changes, suggesting that the altered gait control demands and associated cortical processes can be identified. This and further studies may help to lay the foundation for protocols assessing the cognitive demand of natural gait on the motor system.

## Introduction

Walking while performing a concurrent task, like talking or typing on a smartphone, is a common dual-task in daily life. Altered gait characteristics, especially reduced gait speed in dual-task conditions (1) may predict cognitive impairment as early as 10 years before its onset (2). Moreover, altered gait speed is associated with structural changes in the brain, for instance in the frontal cortex and the basal ganglia (3). Hence, capturing the neural signature of gait control may provide another sensitive tool beyond assessing gait characteristics alone. The early identification of individuals at risk for the cognitive decline may offer earlier diagnosis and thus enhanced opportunities for disease-modulating interventions. While some aspects of dual-tasking, such as altered gait characteristics, neural correlates of the concurrent tasks, or some neural correlates of gait itself have been investigated, other signatures are less well-understood even though they are commonly used to study single-task gait and are sensitive to gait demands (4–7). In our view, understanding these correlates of single- and dual-task walking in healthy individuals will provide another important step toward further understanding patterns of healthy aging and dissociating them from those that reflect an increased risk for neurodegeneration.

While gait is often viewed as an automatic activity, it has also been shown to require some level of cortical control. Studies have shown altered gait patterns while dual-tasking compared to single-task walking (8). Moreover, neural correlates of gait have been discovered in various cortical areas, such as the pre-supplementary and supplementary motor area, as well as central sensorimotor and posterior-parietal regions [for a review see (9)]. Walking is an important activity of everyday life and can be impaired due to various neurodegenerative diseases, like Parkinson's disease (10). This has been linked to a loss in quality of life (11, 12). Hence, understanding neural correlates of gait is vital.

Dual-task effects (DTEs) on gait, such as decreased speed, decreased cadence, decreased stride length, increased stride time, and stride time variability have been observed repeatedly (8). Several theories aim at explaining why DTEs emerge. According to *multiple resource models*, the performance of a task demands specific resources. Correspondingly, tasks only interfere with each other if they demand common, limited resources (13, 14). DTEs on gait will thus only arise if a concurrent task involves areas also needed for gait control. DTEs may manifest in the gait performance, the concurrent task performance, both, or neither (15). Their manifestation may be influenced by the participant's task prioritization (16). Both, performing cognitive tasks - especially ones using internal reference compared to external frames - as well as complex motor tasks while walking, alter gait patterns (8, 17, 18).

Gait characteristics change when walking on uneven compared to even terrain (19). Prominent findings are slower stride time and greater stride time variability on uneven terrain compared to even terrain during unconstrained overground walking (20, 21). When walking speed is constrained, stride times and steps are shorter during perturbed compared to normal treadmill walking (22, 23). These adaptations are assumed to be compensatory, supporting gait stability and balance (20). They are associated with greater metabolic cost (24, 25). Still, decreased gait stability has been reported for some terrains (26) and is linked to greater cognitive demands leading to altered task prioritization (16) and potentially greater DTEs (27).

Several studies investigated neural correlates of a concurrent task performed while walking compared to sitting or standing [(28–32), for a review see (33)]. It has been investigated, whether these correlates change with aging [for a review see (34)]. In addition, electroencephalography (EEG) power differences between single and dual-task conditions have been observed frequently (35–37). For instance, EEG alpha power was found to be reduced at central locations while performing a concurrent motor task compared to single-task walking (17). Moreover, the performance of concurrent cognitive task results in alpha and beta power reductions at central and frontocentral sites for dual- compared to single-task walking (17). In addition, gamma power increases over frontal areas during finger tapping while walking compared to walking alone (18). Finally, neural correlates of gait can be defined as gait-cycle-related power fluctuations. Specifically, gait-cycle-related spectral perturbations (gait ERSPs) can be calculated as spectral perturbations relative to a standing baseline. Whereas, gait-phase related spectral power modulations (GPMs), are gait ERSPs further baseline corrected to the mean power at each frequency across the average gait cycle (38) (for details see Time-frequency decomposition). GPMs thus show power modulations over the time of gait-cycle. GPMs are altered by dual-tasking (7, 39). So far, few studies investigated GPMs in dual-task gait. In one study, GPMs were only altered by some concurrent tasks. GPMs changed by talking to the experimenter but not by typing on a smartphone (7). This suggests that some secondary tasks draw more on gait-related resources than alternative tasks.

Neural correlates of gait are not only modulated by concurrent tasks but also by terrain. Compared to walking over an even surface, differences in central sensorimotor and parietal clusters have been observed during ramp and stair ascent (6). In both clusters and both conditions, a greater beta desynchronization was evident during initial double support compared to walking over even terrain, which may indicate greater cortical recruitment to tackle increased task difficulty (40). Furthermore, in the central cluster, a greater alpha desynchronization has been reported at the same time. For stair ascent, a greater gamma synchronization has been reported during the first swing phase (6). Taken together, these results suggest that navigating more complex terrains poses greater cognitive demands on cortical gait control, which can be observed in altered GPMs. Neural correlates of a cognitive task are altered by standing compared to walking and by terrain complexity (29). Yet, it remains open whether potential influences of a concurrent task and terrain complexity on GPMs interact.

In the present study, we investigate whether the gait-phase-related neural activity is altered by terrain and whether an interaction between terrain and the performance of a concurrent task can be observed. We asked participants to walk two different routes across campus: over an even, paved terrain (even) and an uneven lawn (uneven). Both conditions were performed alone (single-task, ST) or while performing a concurrent motor task (self-paced button pressing, dual-task, DT). We employed mobile EEG to study GPMs during these conditions. GPMs were chosen because they may reflect adaptive cortical contributions to gait control and execution. We used overground walking because it has higher ecological validity than treadmill walking. A simple finger-tapping task was selected as a concurrent task to simulate tapping on a smartphone, similar to a previous study (7). As task and terrain effects were reported previously (6, 7), we compared gait-related activity at various frequencies and time points at an electrode regularly associated with gait control and possibly capturing the cortical activity of motor and premotor areas (i.e. Cz) (5, 7, 17, 41–45). Furthermore, we investigated whether gait performance (i.e., stride time and stride time variability) changed with terrain and dual-tasking. We expected longer stride times and greater stride time variability on uneven compared to even terrain [(20, 46), for a review see (47)] and during dual- compared to single-task gait (17).

Since mobile EEG data can be contaminated by various artifacts, we first assessed whether sufficient data quality could be obtained after artifact attenuation. Therefore, the multidimensional gait-related artifact footprint approach (48) was used to validate mobile EEG signal quality independent of the research question at hand.

## Materials and methods

### Participants

A young, healthy, right-handed sample of *N* = 26 participants (19 females and seven males) with unimpaired gait was recruited via the online platform of the University of Oldenburg. Participants provided written informed consent and received monetary compensation (10€/h). The study was approved by the ethics committee of the University of Oldenburg (permit number: 2018-079).

### Materials

EEG data were captured using two Live Amps (Brain Products GmbH, Gilching, GER) placed on top of participants' heads capturing data of 66 Ag/AgCl passive electrodes embedded in custom 64-channel caps (Easy Cap GmbH, Herrsching, GER). Wires were bundled to reduce their motion and associated data noise (31, 49). Electrode impedances were decreased to at least 10 kΩ. The online reference was FCz, the ground AFz, and the sampling rate was 500 Hz [for a setup picture and channel layout see (48)]. EEG data were streamed via a Bluetooth connection using the LiveAmpConnector software (version 1.16, bit.ly/31P2mrd). Participants held the recording laptop (Ultrabook, Latitude 5289, Dell Inc., Round Rock, TX) in tablet mode with both of their hands. Presentation software (version 20.02, Neurobehavioral Systems, Inc., Berkeley, CA, RRID:SCR_002521) was used to control the experiment. Participants' motion was captured by a 3D accelerometer built into the EEG amplifier on top of their heads, and two 3D accelerometers (eMotion Faros 180°, Mega Electronics Ltd, Kuopio, FIN) placed on top of their shoes. EEG data and experimental events were time-synchronized using Lab Streaming Layer and stored in a file by the LabRecorder software (version 1.13, bit.ly/2ULAFhb). Foot acceleration sensor signals were time-synchronized to the EEG data offline using synchronization triggers sent at the beginning and end of the recording [as described in (42)]. This dataset was analyzed in a previous publication (48) and is available at OpenNeuro (see Data Accessibility Statement).

### Procedure

**Figure 1:**
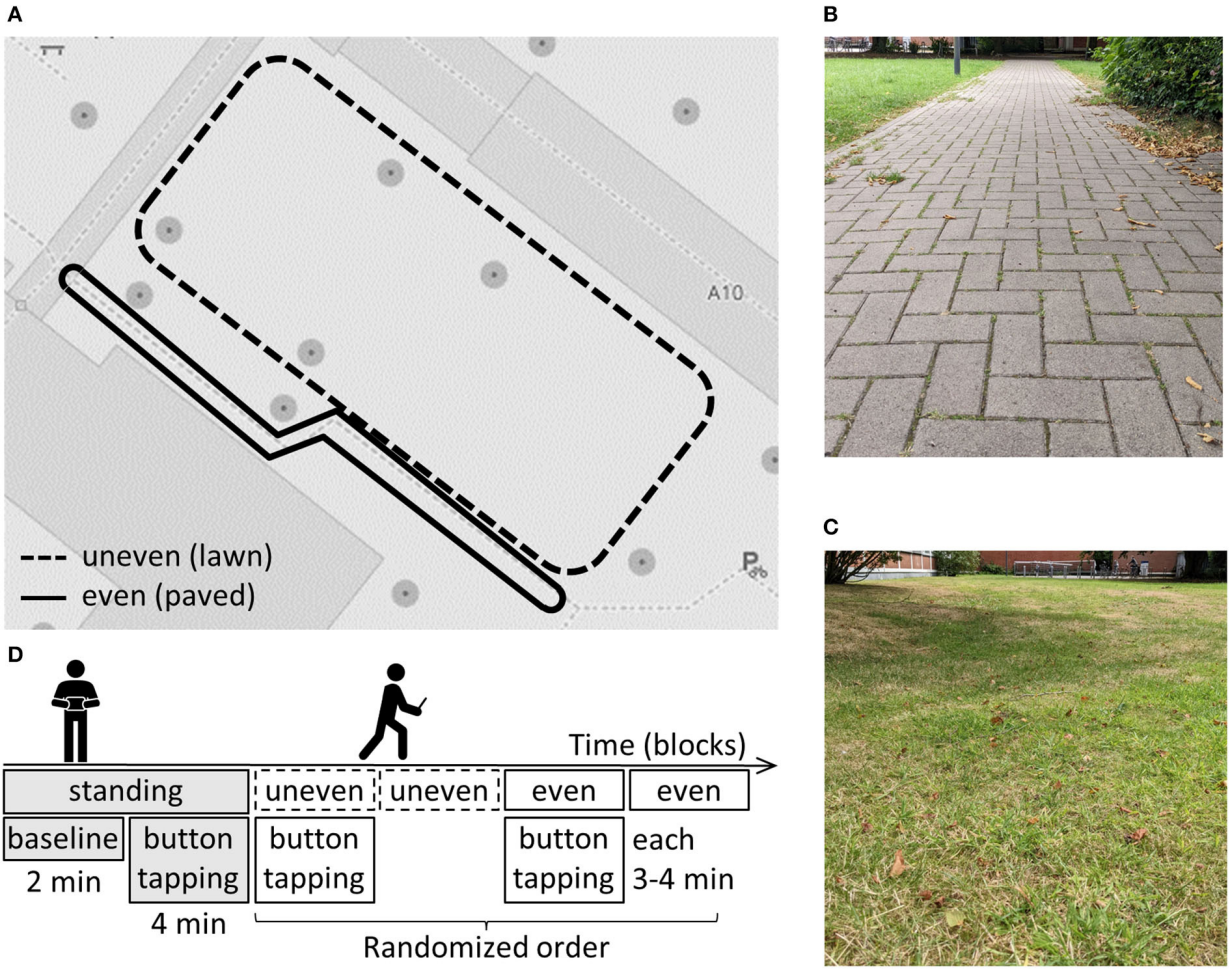
**(A)** Map of the two routes with different terrain **(B)** picture of the even terrain **(C)** experimental structure outdoors **(D)** picture of the uneven terrain.

Data were recorded during a gait-initiation task indoors and an overground walking task outdoors. Only the EEG data recorded outdoors was analyzed in this study. Outdoors, a 2-min standing baseline was followed by a self-paced button-pressing task for 4 min (see Figure 1B). Participants were instructed to press buttons displayed on the left and right sides of the laptop's touchscreen with their left or right thumb, respectively. They were asked to surprise the experimenter with the hand and time of the button-presses and to wait for 1 to 3 s after each button-press until the next one. This elicited a movement-related cortical potential (50), as analyzed in a previous study (48). Participants were asked to fixate their gaze to a point at eye level, and not to look down at the laptop. So, to inform them about successful button presses, each button press was confirmed with a brief sound. Subsequently, participants were shown two different routes (see Figure 1A) next to each other on the university campus. One was over lawn (uneven terrain, see Figure 1C) and the other one a paved footpath (even terrain, see Figure 1C). Both routes were marked with pylons and had to be walked in a clockwise direction. As the uneven route was slightly longer, subjects walked it two times while walking the even route three times, resulting in 3 to 4 min of walking per condition. Both terrains were walked two times in a randomized order: once with the concurrent performance of the button-pressing task (DT) and once without (ST), resulting in four conditions (see Figure 1B). Participants were instructed to avoid superfluous head movements and jaw clenching during the blocks. After completing the experiment, participants were asked to indicate on a 5-point Likert scale how difficult (1 = not difficult at all, 5 = very difficult) they perceived both terrains (even, uneven) to walk on.

### Gait analysis

Gait events were identified with custom scripts in MATLAB (MathWorks Inc., Natick, MA, version R2021a, RRID:SCR_001622) using the data from the two 3D accelerometers placed on top of participants' shoes. Accelerometer data of each foot was processed independently. First, data were imported at 250 Hz. Second, they were detrended and low pass filtered with a second order, zero-phase, infinite impulse response Butterworth filter with a cut-off of 30 Hz. An interim dataset was low-pass filtered at 6 Hz. Only data captured during walking bouts was used for step detection. The interim data (low-pass filtered at 6 Hz) was only used to determine the number of steps in each walking bout. To do so, peaks in the vertical acceleration exceeding 0,6 g with a minimal distance of 500 ms were identified. This marks roughly mid-swing. This threshold had to be adapted to 0,24 g for one subject. The 30 Hz low-pass filtered data was used to detect heel strike and toe-off. The first peak exceeding 0,6 g following a step marker was determined to be a heel-strike. Toe-off was determined as the mean of the two highest peaks in anterior-posterior acceleration exceeding 0,2 g in the 500 ms preceding a step marker. All peaks were identified using the MATLAB function *findpeaks* ().

This gait detection approach was validated with a motion capture system (Vicon, Oxford, UK). An average temporal error of around 2 ms was observed and considered acceptable.

Only gait cycles in which (1) the order of gait events was right heel strike (RHS), left toe-off, left heel strike, right toe-off, RHS and (2) the next RHS occurred between 0.5 and 1.5 s after the previous one, were deemed plausible and were analyzed further.

Stride times of all remaining strides were calculated as the time from one RHS to the next (in seconds). Stride time variability was calculated as the coefficient of variation (in percent) by dividing the standard deviation of the stride times by the mean stride time and multiplying the result with 100 (17).

### EEG pre-processing

EEG data were processed in MATLAB (MathWorks Inc., Natick, MA, version R2021a, RRID:SCR_001622) using EEGLAB (51) (version 2020.0, RRID:SCR_007292), and Brainstorm (52) (version Jan 2020, RRID:SCR_001761) toolboxes, as well as custom code. The scripts are accessible via GitHub (see Data Accessibility Statement).

**Figure 2:**
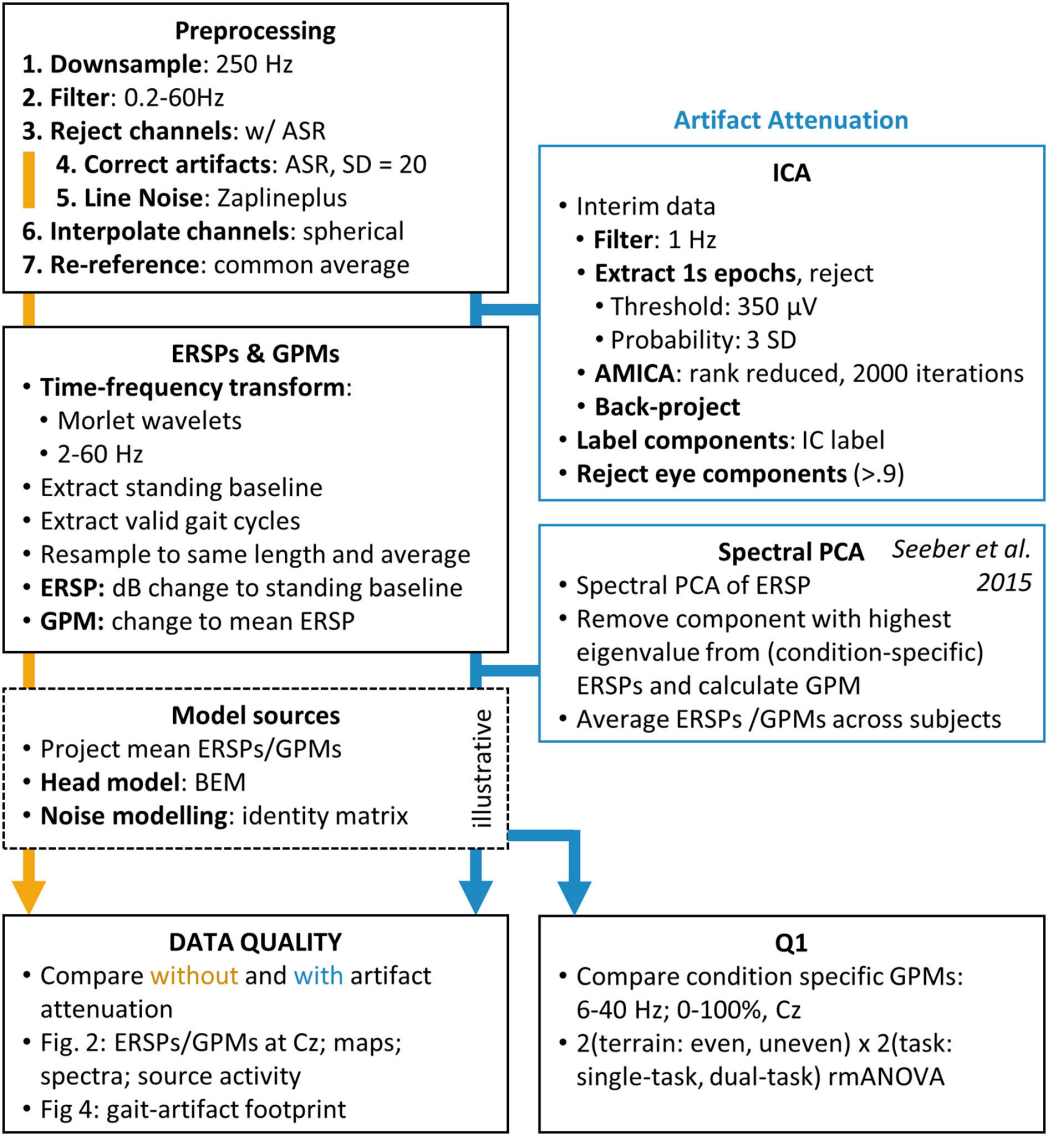
EEG preprocessing and artifact attenuation pipeline.

First, data were downsampled to 250 Hz and then filtered between 0.2 Hz (passband edge, order 4,126) and 60 Hz (passband edge, order 56) with zero-phase, finite impulse response (FIR) filters. Channels were rejected using *clean_rawdata* (flatline channels for 5 s, channel correlation below 0.8 and line noise above 4, other parameters disabled, version 2.3). Artifact subspace reconstruction (53), was calibrated with a standing baseline at the beginning of the recordings and used to correct artifacts with a cut-off of *SD* = 20, following the version's default settings and recommendations (54). Then line noise was corrected with the zapline plus tool (55) (available at https://github.com/MariusKlug/zapline-plus, retrieved 26.10.21, with noise frequency 50 Hz, highest Frequency 61 Hz) an extension to zapline (56). After spherical interpolation of the previously rejected channels, channels were re-referenced to the full rank common average (version 0.10, available at http://sccn.ucsd.edu/eeglab/plugins/fullRankAveRef0.10.zip). To further attenuate artifacts we combined two established methods of gait-artifact attenuation, notably independent component analysis (ICA) to attenuate eye artifacts and spectral principal component analysis (sPCA) (57) to attenuate muscle artifacts. For an outline of the EEG preprocessing see Figure 2. This approach was chosen after carefully comparing different approaches, which are described in detail in the Supplementary material.

#### Independent component analysis

For ICA decomposition, an interim dataset was high-pass filtered at 1 Hz (passband edge, order 415) and consecutive 1-s epochs were extracted. Epochs with samples exceeding a threshold of 350 μV or a joint probability of three standard deviations (*jointprob*) were rejected. The remaining data were decomposed using adaptive mixture independent component analysis (AMICA, 1 node, 2 threads, 1 model, 2000 iterations, version 1.5.2) (58). The obtained weights were back-projected to the original dataset. AMICA decomposition is often chosen for gait EEG (5, 39, 40, 59–69) because it attenuates EMG artifacts of treadmill walking better (70) and produces more near-dipolar ICs and a greater mutual information reduction than other ICA algorithms (71). Afterward, all ICs were automatically classified using IClabel (72) (version 1.3), and components exceeding a 90% probability of representing eye movement or blinking artifacts were rejected.

#### Time-frequency decomposition

Gait ERSPs were calculated as follows: linearly spaced (2 Hz steps) Morlet-wavelets were used to time-frequency (TF) decompose the data from 2 to 60 Hz. Gait cycles or strides were extracted (RHS to the next RHS). Only gait cycles with plausible order and timing of gait events, as determined in the gait analysis in which none of the samples exceeded 350 μV were kept. Each gait cycle (RHS to RHS) was then linearly resampled to an arbitrary, uniform length of 100 samples using the *resample* function (38, 57) so that each sample corresponded to one percent of the gait cycle. All gait cycles were averaged resulting in one TF map per channel and participant. The power of a standing baseline (4 min) was computed with the same wavelets used for the gait ERSPs and averaged across time. Power change to the standing baseline (decibel, dB) was calculated. Gait ERSPs were calculated for all conditions together and used for the sPCA (see Spectral principal component analysis), as well as for each condition (ST even, ST uneven, DT even, DT uneven) alone. GPMs, are gait ERSPs with another baseline correction. GPMs of each channel were calculated by subtracting the mean gait ERSP power across time from each frequency (38).

#### Spectral principal component analysis

Seeber et al. (57) introduced an sPCA approach to attenuate muscular artifacts in gait EEG. They assumed that the greatest spectral variance of the TF decomposed signal is not of neural origin and can be removed. We implemented the sPCA based on scripts provided by Seeber et al. (57). The sPCA was performed on single-subject gait ERSPs averaged over all gait cycles and conditions and decomposed using a PCA. The resulting eigenvectors were sorted by decreasing eigenvalue and the component with the greatest eigenvalue was rejected. The remaining components were back-projected using a weighting matrix. The same weighting matrix was used for the averaged as well as condition-specific ERSPs, in order not to introduce condition-specific variance by the artifact attenuation.

#### Source modeling

For illustrative purposes, the obtained results, i.e., group averages with and without artifact attenuation, were projected into the source domain. Sources were modeled using the default anatomy provided by Brainstorm and a three-layer boundary element model implemented in openMEEG (73). The channel locations were projected on the scalp. The inversion kernel was obtained following dynamical statistical parametric mapping (74) with a minimum norm estimator, constrained dipole orientations, and an identity matrix for noise modeling. The attained kernel was used to project the group averaged TF maps of all channels with and without artifact attenuation. If differences in condition-specific GPMs were found, condition effects were projected as well (see Gait-phase related power modulations).

### Statistical analysis

Statistical analyses of behavioral data were performed in JASP (75) (version 0.1, RRID:SCR_015823). Neurophysiological data was tested in EEGLAB (51) (version 2020.0, RRID:SCR_007292). Assumptions were checked and tests were adapted if indicated. Appropriate effect sizes were reported: Cohen's d (*t*-test), R (Wilcoxon signed-rank), or partial eta squared (repeated measures (rm)ANOVA). The alpha level was kept at 0.05. *P*-values were corrected for multiple comparisons using Bonferroni–Holm correction.

#### Behavioral analysis

Walking difficulty of the even and uneven terrain, as assessed by subjects' self-reports, were compared with a dependent samples Student's *T*-Test. If differences deviated from normality (*p*_Shapiro − Wilk_ < 0.05), a non-parametric alternative, i.e., the Wilcoxon signed-rank test was chosen.

Gait performance within each condition was compared with a 2 × 2 rmANOVA with the factors *terrain* (even, uneven) and *task* (ST, DT) for the dependent variables stride time and stride time variability respectively.

#### Neurophysiological analysis

##### EEG data quality

To assess the success of the artifact attenuation we compared EEG data with and without (only filtered and downsampled data, channel rejection, and interpolation, and re-referenced to common average) artifact attenuation. Artifact reduction was assessed quantitatively with a previously proposed gait artifact footprint approach (48). The following footprint features were assessed: (B) explained variance across frequencies, (C) lateral to medial channel power ratio, (D) neck channel power ratio, (E) double to single support power ratio, (F) standing to walking power ratio. In contrast to the previous publication, we calculated the features based on the dB power change (vs. power ratio) to a standing baseline since this data will be used for the remaining analyses and adapted the normalization procedures. Furthermore, for two features (C, D) power decreases were discarded. This was done because gait related-artifacts have been associated with power increases compared to a standing baseline, whereas power decreases are associated with neural activity (e.g., alpha and beta band) (76) and the features aim to quantify artifact extent. The code for the footprint calculation has been updated accordingly^1^ The footprint was calculated with and without artifact attenuation and the Euclidean distance between each subject's footprint feature vectors was calculated. Whether this distance was greater than zero was assessed with a one-sample *T*-Test or in case of a deviation from normality (*p*_*Shapiro*−*Wilk*_ < 0.05), with a Wilcoxon signed-rank test.

To assess the presence of neural signals and artifacts associated with gait, we compared group average gait ERSP and GPM with and without artifact attenuation qualitatively at a channel regularly analyzed in gait EEG studies, i.e., Cz (5, 7, 17, 41–45). After artifact attenuation, we expected to recognize neural correlates of gait. For gait ERSPs, alpha and beta power decreases compared to a standing baseline have been observed (7, 57, 63). For GPMs, frequently reported power modulations are beta power decreases during double support and beta power increases during single support (5, 57, 77). Topographies of averaged gait ERSP beta (here: 20–30 Hz) power and averaged absolute GPM beta power with and without artifact attenuation are compared. We expect to reveal spatial patterns associated with artifacts (e.g., increased absolute beta power at lateral channels, associated with EMG activity) without artifact attenuation and patterns linked to neural control of gait (e.g., decreased beta power over central sensorimotor areas) with artifact attenuation.

##### Gait-phase related power modulations

Three nonparametric cluster-based, dependent samples permutation tests (78) were used to compare GPMs from 6 to 40 Hz at Cz of all four conditions (ST even, ST uneven, DT even, DT uneven). The main effects of *task* and *terrain* were assessed by comparing the average GPM of each factor-level with a nonparametric cluster-based, dependent samples permutation test. For instance, the main effect of terrain was assessed by comparing each subject's mean of *ST even* and *DT even* to the mean of *ST uneven* and *DT uneven*. To test the interaction, the paired differences of the main effects *task* and *terrain* were submitted to a non-parametric cluster-based, dependent samples permutation test. Non-parametric cluster-based permutation tests were chosen because their alpha error correction is physiologically plausible. It favors larger clusters extending over several TF duplets. Even local effects may be smeared in time and/or frequency by the wavelet decomposition (79).

In case of significant differences, each cluster's effect size was computed across the average of the cluster by extracting all TF duplets of the condition differences that were part of a cluster and averaging them for each participant separately. Then Cohen's d was calculated with this measure.

## Results

Seven datasets had to be excluded from the analysis due to problems with the recording software (*n* = 5), synchronization triggers (*n* = 1), and not meeting the inclusion criteria (*n* = 1) (48). Data from *n* = 19 participants (age 25 ± 4 years, 13 females, six males) were analyzed.

### Behavioral

#### Terrain

Participants reported that the uneven terrain (lawn) was more difficult to walk on (median, *Mdn* = 2) than the even terrain (paved, *Mdn* = 1, *T* = 171, *p* < 0.001, *R* = 1).

#### Gait performance

**Figure 3:**
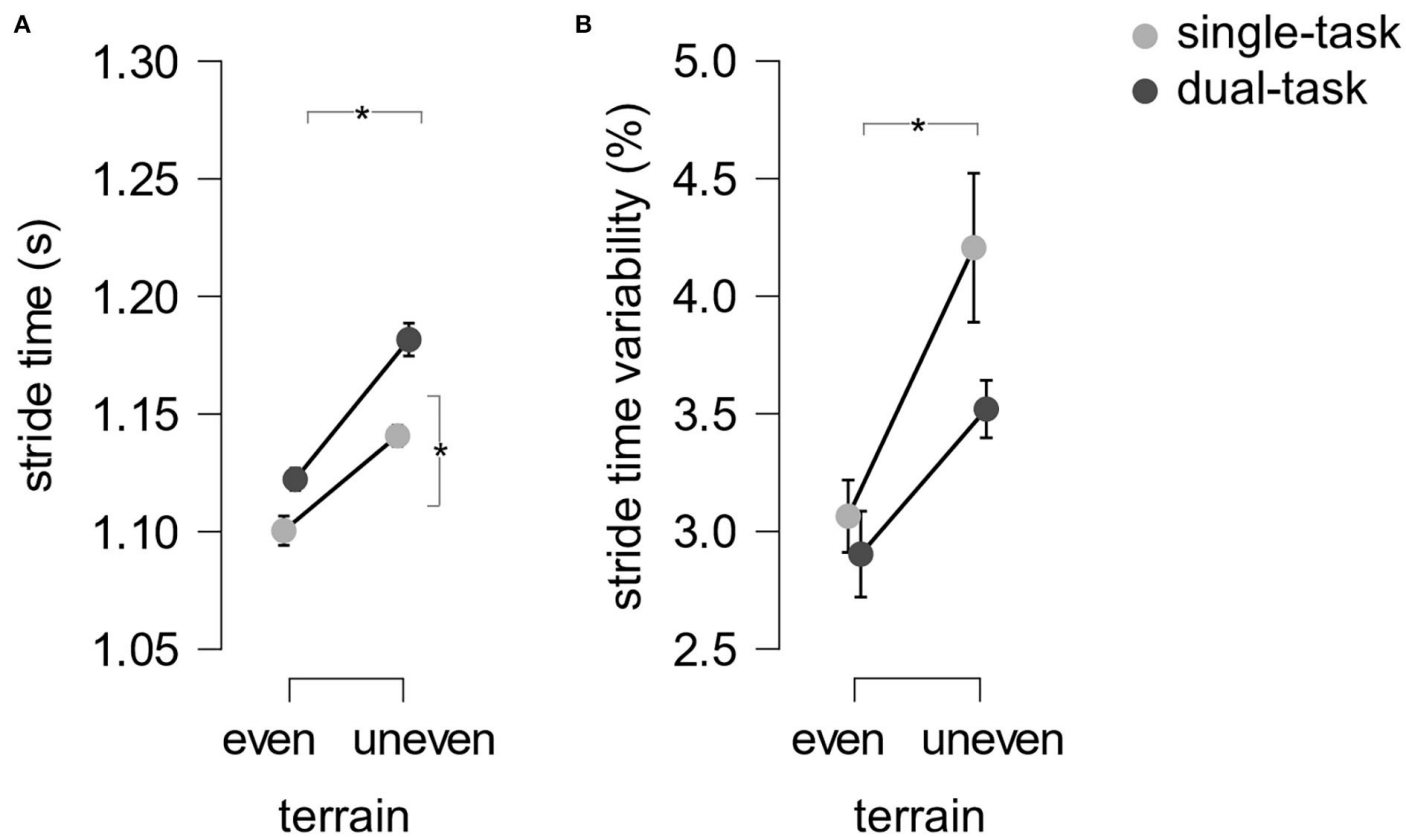
Mean stride time **(A)** and stride time variability **(B)** across subjects. Error bars indicate the standard error of the mean.

*Stride time*. Statistical analysis with a 2 (terrain: even, uneven) × 2 (task: ST, DT) rmANOVA identified main effects of terrain (*F* (1.18) = 102.15.21, *p* < 0.001, η_p_^2^ = 0.85) and task (*F* (1.18) = 27.10, *p* < 0.001, η_p_^2^ = 0.60) but no interaction between terrain and task [*F*(1.18) = 2.48, *p* = 0.133, η_p_^2^ = 0.12, see Figure 3A] for the dependent variable stride time. Participants walked slower on uneven than even terrain (*d* = −2.32) and during double compared to single-tasking (*d* = −1.19).

*Stride time variability*. A 2 (terrain: even, uneven) × 2 (task: ST, DT) rmANOVA exposed a main effect of terrain (*F* (1.18) = 4.68, *p* < 0.001, η_p_^2^ = 0.47) but no effect of task (*F* (1.18) = 3.62, *p* = 0.073, η_p_^2^ = 0.17) and no interaction between terrain and task were found (*F* (1.18) = 1.31, *p* = 0.160, η_p_^2^ = 0.11, see Figure 3B) with the dependent variable stride time variability. Participants' stride time variability was smaller on even than on uneven terrain (*d* = −0.95).

### EEG preprocessing

On average two channels (range: 0 to 5) were removed and 58 independent components (range: 54 to 61) were kept per participant. On average 209 gait cycles were detected per participant and condition (ST even: mean, *M* = 227, range: 202 to 281, DT even: *M* = 235, range: 206 to 285, ST uneven: *M* = 189, range: 160 to 291, DT uneven: *M* = 188, range: 160 to 234). After excluding artifactual gait cycles, on average 193 gait cycles remained per participant and condition (ST even: *M* = 211, range: 70 to 282, DT even: *M* = 225, range: 92 to 286, ST uneven: *M* = 171, range: 52 to 247, DT uneven: *M* = 166, range: 23 to 235).

### EEG data quality

**Figure 4:**
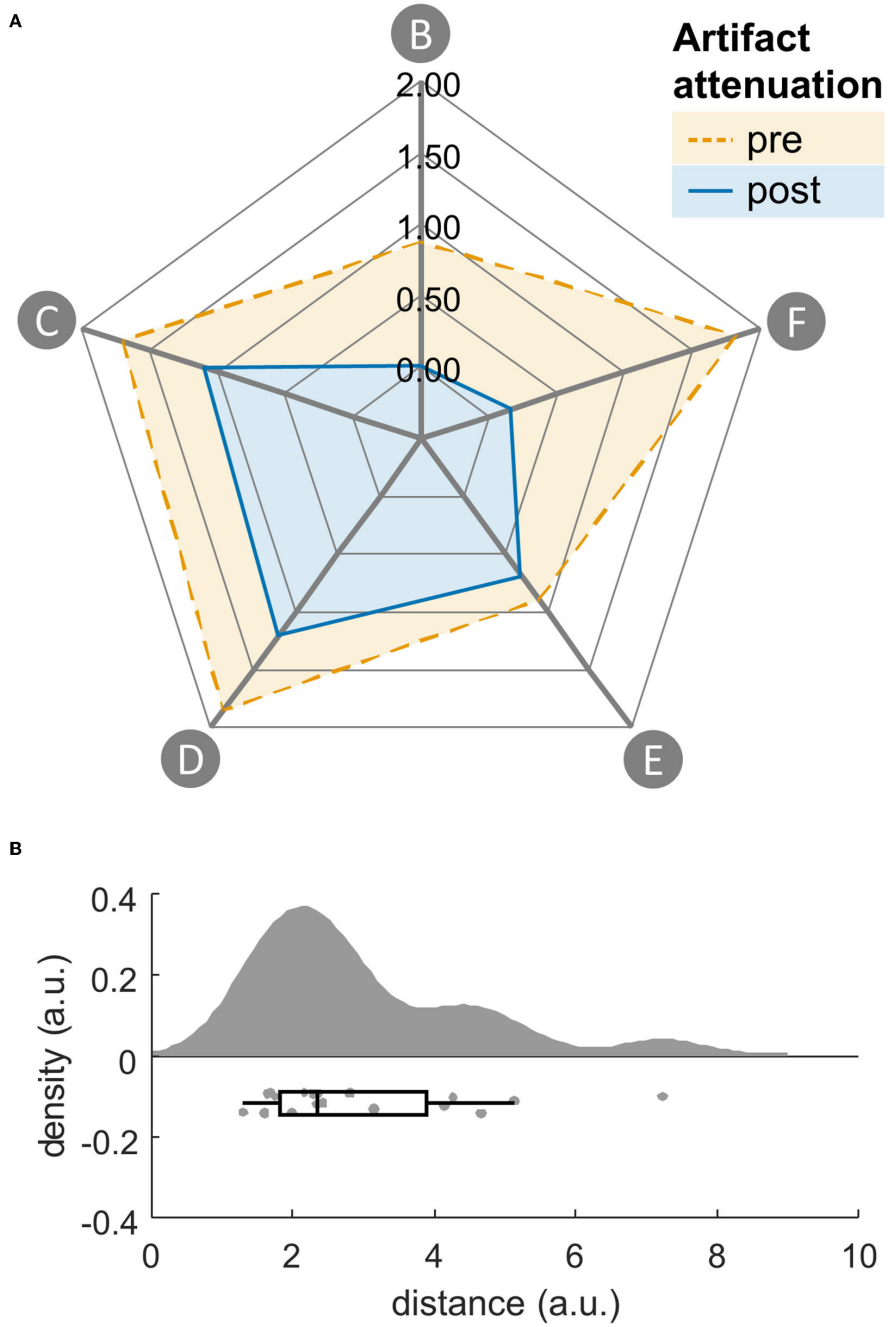
**(A)** Gait artifact-related footprint without (dashed) and with (solid) artifact attenuation with the following features: (feature B) explained variance across frequencies, (feature C) lateral to medial channel power ratio, (feature D) neck channel power ratio, (feature E) double support power ratio, (feature F) standing/walking power ratio. **(B)** Raincloud plots (80) of euclidean distances of footprint feature vectors with and without artifact attenuation. Single subjects represented by dots.

Descriptively, all footprint features decreased due to artifact attenuation. Distances of the footprint feature vectors were greater than zero (Mdn = 2.94, T = 190.00, *p* < 0.001, *R* = 1, see Figure 4).

**Figure 5:**
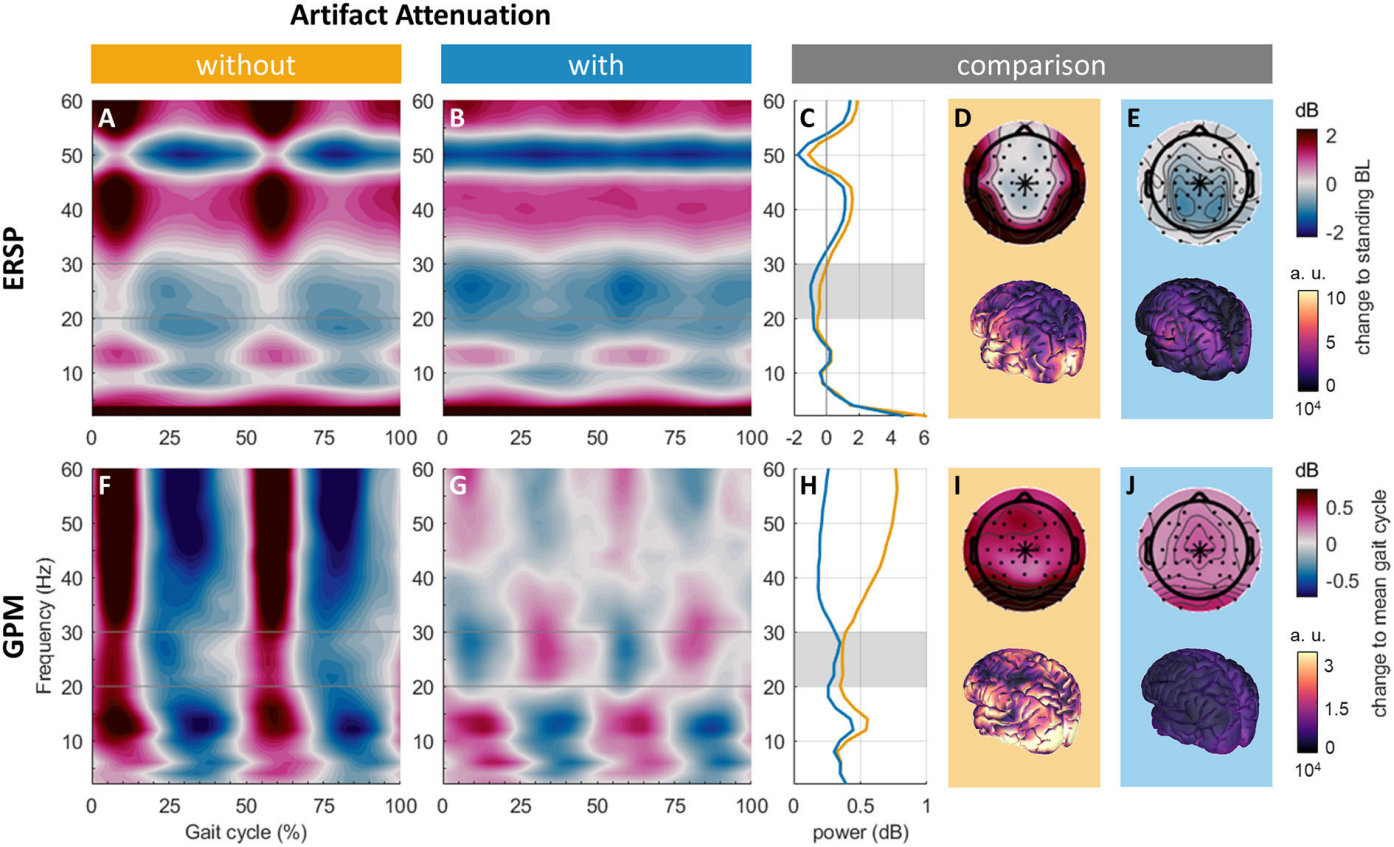
ERSPs at Cz without **(A)** and with **(B)** artifact attenuation. **(C)** Comparison of ERSP spectral power at Cz without (orange) and with (blue) artifact attenuation. Mean beta power (20 to 30 Hz, across the gait cycle) topographies and source activity without **(D)** and with **(E)** artifact attenuation. GPMs at Cz without **(F)** and with **(G)** artifact attenuation. **(H)** Comparison of GPM absolute spectral power at Cz without (orange) and with (blue) artifact attenuation. Mean absolute beta power (20 to 30 Hz, across the gait cycle) topographies and source activity without **(I)** and with **(J)** artifact attenuation.

Qualitatively, gait ERSPs and spectra looked similar with and without artifact attenuation. Both gait ERSPs showed decreased power from ~8 to 12 Hz, 18 to 32 Hz, and around 50 Hz. Broadband activity during double support (~0 to 15% and 50 to 65%) was reduced by artifact attenuation (see Figures 5A,B). The power reduction around 50 Hz persisted and may be due to the remaining line noise in the standing baseline (see Figure 5C). Gait ERSP topographies revealed increased power at lateral electrode sites, most pronounced over the neck (see Figure 5D), which disappeared with artifact attenuation uncovering a power decrease at parietal electrode sites (see Figure 5E). This was also reflected by the source modeling showing activations at temporal and occipital regions that were reduced with artifact attenuation while a much smaller activation in the parietal cortex persisted (see Figures 5D,E).

GPMs at Cz showed broadband power increases during double supports (approx. 0 to 15% and 50 to 65%) and power decreases during single support (see Figure 5F). After artifact attenuation, this broadband activity was replaced by alternating patterns of power changes: during double support, power increased from approximately 4 to 16 Hz and 42 to 60 Hz and decreased from 18 to 40 Hz (see Figure 5G). This pattern was reversed during single support, i.e., power decreased from ~4 to 16 Hz and 42 to 60 Hz and increased from 18 to 40 Hz. The spectra showed power reductions over 30 Hz by artifact attenuation, while the power reduction below 30 Hz was negligible (see Figure 5H). Topographies revealed that power at electrodes located over the neck was reduced but not fully diminished by artifact attenuation (see Figures 5I,J). With artifact attenuation, activity slightly anterior to the vertex emerged in the topography (see Figure 5I). The source reconstruction revealed activity all over the cortex, especially over occipital areas, which were reduced by artifact attenuation

### Gait-phase related power modulations

**Figure 6:**
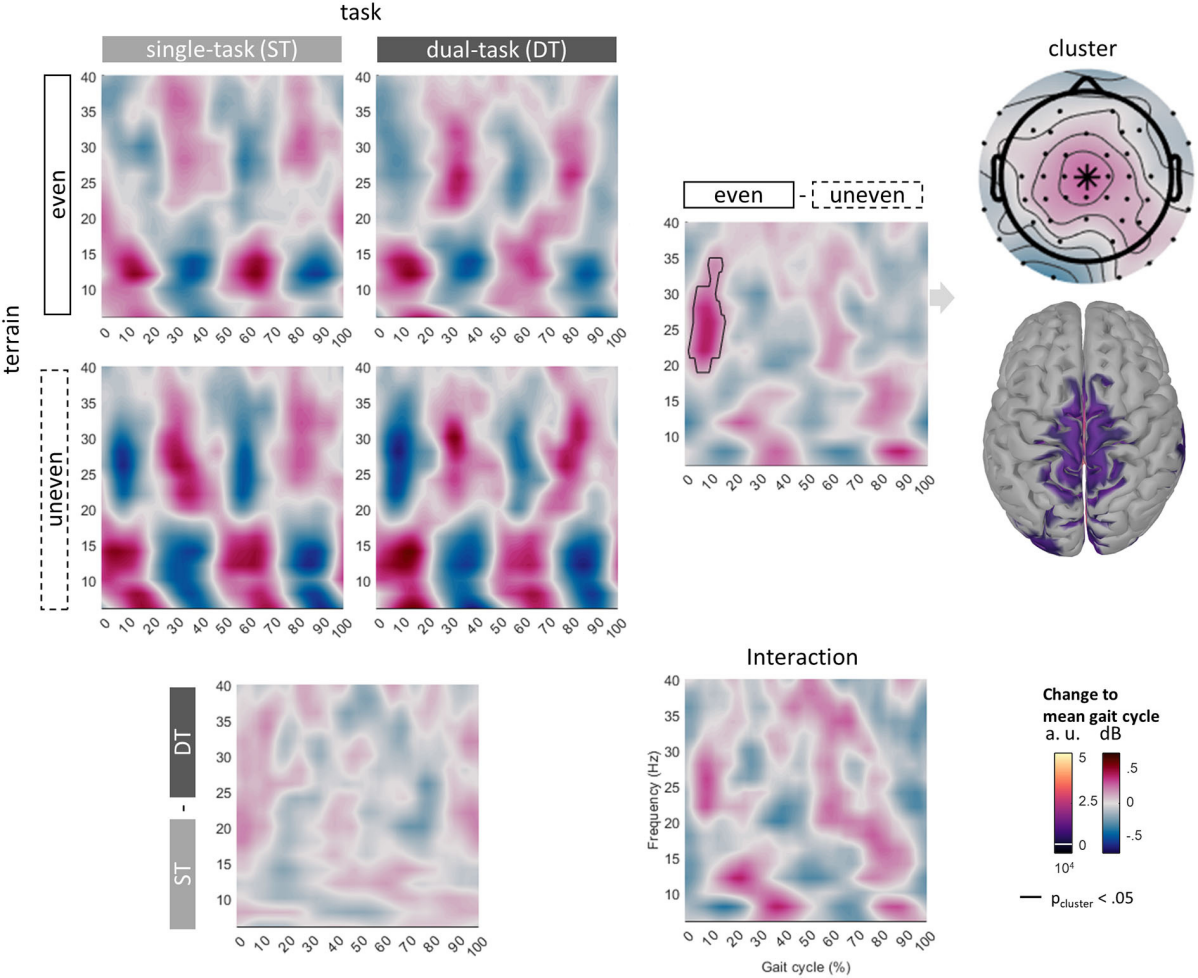
Grand average GPM at Cz of all conditions from 6 to 40 Hz across the whole gait cycle (0% initial contact right to 100% next initial contact right). Cluster uncovered with nonparametric permutation tests marked with a solid outline. Topography and projected sources of the cluster mean on the right side.

GPMs at Cz showed patterns of alternating power modulations from 6 to 40 Hz across different frequency bands in all conditions. Theta (6 to 8 Hz) and alpha/mu (10 to 16 Hz) GPMs increased approximately during double support (0 to 16% and 50 to 66%) and decreased during single support with the theta modulation being slightly later. Beta (20 to 40 Hz) GPMs decreased during double support and increased during single support. Descriptively, power modulations were greater while walking over the uneven compared to the even terrain. Cluster-based non-parametric permutation tests revealed an effect of terrain but neither of the task nor an interaction. GPMs of walking over an even compared to an uneven terrain were significantly different. As non-parametric permutation tests do not allow the interpretation of the location of the cluster (see Figure 6, solid outline), we were not able to make any further conclusions in this study but described the cluster to inform future studies. Descriptively, a greater power decrease over uneven than even terrain was observed in a cluster ranging from 18 to 34 Hz at 2 to 15% of the gait cycle. The average cluster effect was *d* = 1.22. The topography of the cluster showed the greatest effect of terrain around the vertex. Source modeling also indicated activity at central sensorimotor areas below these electrode locations. Yet, the greatest activity was estimated to originate from the lingual gyrus and isthmus (not visualized).

## Discussion

We compared gait performance and the associated neural correlates in young, healthy participants walking over two different terrains outdoors (even: paved; uneven: lawn) with and without performing a concurrent task (self-paced button tapping). Participants perceived the uneven terrain as more difficult to walk on than the even terrain. Gait performance, i.e., stride time and stride time variability changed with terrain, but only stride time also changed for single- compared to dual-task walking. No interactions were observed. After ensuring that sufficient EEG data quality was achieved by artifact attenuation, we compared GPMs at Cz and observed a main effect of terrain. First, the effects of terrain and task on gait characteristics will be examined, followed by the EEG signal quality achieved with artifact attenuation. Then the impact of terrain and task on gait-related neural activity is discussed. Finally, the implications of this study are summarized.

### Gait performance

Following our hypothesis and previous studies, stride times were shorter on even compared to uneven terrain (20, 21) and in single- compared to dual-task conditions (8, 17, 18), but no interaction was observed. Contrary to our expectations, no effect of task on stride time variability was found.

As demonstrated in previous studies, stride times were longer and more variable on uneven compared to even terrain (20, 21, 46). These adaptations have been linked to increased stability and higher metabolic cost (24). The gait changes observed in this study go hand in hand with the greater perceived difficulty of the uneven terrain, even when both terrains were rated as easy to walk on by the young, healthy participants.

DTEs on gait performance have been reported already in young, healthy adults [for a review see (8)]. We could replicate longer stride times but did not observe a greater stride time variability during dual- compared to single-task gait. This is surprising as DTEs on stride time variability are estimated to be greater than DTEs on stride time, at least for concurrent cognitive tasks (8). The effect on stride time variability was observed with complex motor tasks, and simple cognitive tasks with a less demanding motor component, such as pressing a button on target tones (17). Interestingly, DTEs depend on walking modality. They have been observed during overground walking while being absent in treadmill walking, although participants did not report differences in the perceived task difficulty (81, 82). This stresses the importance of investigating more ecologically valid overground walking as well. Thus, we hypothesize that altered stride time variability may have been observed with a concurrent task placing a greater demand on cognitive resources also needed to control gait resulting in greater dual-task interference (13, 14, 16, 27). The lack of interaction between stride time and stride time variability may indicate that the chosen task and terrain affect gait characteristics independently.

As we did not ask the participants to rate the perceived difficulty of the button-pressing task or cognitive demands [cf. (28, 29)], we could not evaluate task difficulty of all conditions and relate it to the obtained measures.

Moreover, a more detailed kinesiological analysis was not possible with our gait detection. We could only investigate temporal gait parameters, but not spatial ones like step width and length, toe clearance, the center of mass, or spatiotemporal ones like speed which also change with uneven terrain walking (20, 83). DTEs are particularly well-captured by gait speed, especially in young, healthy adults (84). Moreover, gait speed is a sensitive marker to discriminate between healthy and neurological subgroups (8). Using more sophisticated inertial measurement units, which also contain gyroscopes and magnetometers, instead of just accelerometers, most of these measures can be collected during overground walking, even outdoors (24). Yet, this simple setup already shows changes in some gait characteristics and showcases the possibility to detect such changes with sparse setups that could be integrated into clinical practice more easily (85–87).

### EEG data quality

EEG data quality improved considerably with artifact attenuation. Patterns previously associated with gait-related artifacts were greatly diminished. The broadband synchronization compared to a standing baseline during double support at Cz disappeared with artifact attenuation (see Figures 5A,F) (76). Moreover, power at lateral electrodes decreased with artifact attenuation (see Figures 5D,I) (48). Accordingly, the gait-artifact footprint decreased (see Figures 5D,I).

Still, some residual artifacts remained. This is for example highlighted by the high beta modulation depth at electrodes located over neck muscles, which remained after artifact processing (see Figure 5J). As previously shown with this and other datasets (43, 48), the artifact extent around the vertex was not as widespread as initially observed (88). However, while motion artifacts typically increase with gait speed (43, 67, 89) we found greater modulation depth in conditions of slower gait. Hence, we conclude that the observed condition difference did not arise due to residual motion artifacts but represents altered brain activity.

We compared several preprocessing pipelines (see Supplemental materials) [for a review on possible artifact attenuation strategies for mobile EEG data recorded during motion see (69)] and opted for an sPCA following artifact subspace reconstruction and ICA. This approach requires no additional parameters beyond deciding on the TF transformation. Moreover, it is computationally less expensive and reproducible compared to, for instance, the k-means clustering of independent components around a random seed as implemented in EEGLAB. SPCA can be performed on the sensor or in the source space (38, 57). It assumes that the first principal component, explaining the greatest spectral variance, is likely related to muscle artifacts, and can be removed without affecting neural signals. This approach allows single-subject analysis since no data aggregation over several subjects is necessary. In previous work, sPCA has yielded promising results with treadmill walking and wired EEG transmission (38, 57). In our study, the use of sPCA contributed to good EEG signal quality in overground walking and wireless EEG transmission. Future research may investigate whether these findings generalize further. Because of these advantages, we decided to use an sPCA, although repetitive clustering of dipolar sources yielded very similar results (see Supplemental materials). The repetitive clustering approach allows finding a best-fitting cluster to an a-priory chosen MNI coordinate by repeatedly clustering (and thus mitigating the effect of random seeds in k-means clustering) and ranking the obtained results with a composite score of various features (90). It is implemented in the BeMoBIL pipeline (91) (available at https://github.com/BeMoBIL/bemobil-pipeline). This approach may be better suited for confirmatory analysis of group data with effects located at an a priori known region.

We suppose that greater spatial coverage, as well as digitized electrode locations and subject-specific structural MRI, would improve artifact attenuation further. To understand neural correlates of gait further, studies including these additional measures will be of great significance, however, in line with previous studies (6, 7, 92), our approach demonstrates that gait-related neural activity can be investigated with small setups [for system recommendation see e.g., (93)] with measurements from few additional sensors. These studies using less equipment are needed to investigate possible translations into clinical practice, where less-costly and time-consuming setups are of relevance.

### Gait-phase related power modulations

GPMs looked qualitatively similar across all conditions. This may indicate that the underlying neural control remained predominantly unchanged. Patterns of alternating synchronization and desynchronization are linked to certain motion phases of both the left and right sides. In gait, this is for instance the beta ERD at the beginning of swing phases, both directly before right and left toe-off. These patterns are distinct for different frequency bands and have been linked to transition phases, such as the switch from stance to swing phases during walking, but also the switch from extension to flexion in other periodic movements like finger-tapping or cycling (45, 94). Especially for beta power, these ERDs may be linked to the higher demands of cortical control during these transitional phases (95–97).

Descriptively, modulation depth at Cz was greater for uneven terrain than even ones. This is especially pronounced in the alpha/mu and beta bands. As subjects rated the uneven terrain to be more difficult to walk on, this may be linked to greater cortical control for more difficult tasks. Greater alpha/mu and beta ERDs may indicate increased activation of underlying cortical networks as the physical requirements and/or complexity of the task rise (6). Especially beta desynchronization has been linked to sensorimotor integration and error monitoring required to maintain performance (7, 17, 39, 40, 61, 68). Previous research has suggested that the right heel strike may indicate a starting point of a gait cycle and hence is more demanding than other gait events as reflected by alpha and beta ERDs localized to sensorimotor areas (6). Complex terrains may challenge gait stability (19). Similarly, increased gait stability (by restricting participants' medio-lateral movements) resulted in reduced beta ERDs compared to normal treadmill gait especially around right heel strikes (98) while modulation depth increased for more complex terrain like steps or ramps (6). Here, GPMs of even and uneven terrain differed at Cz. Descriptively, the beta ERD following RHS was greater on uneven terrain compared to even ones. A greater beta modulation, i.e., greater desynchronization, during walking over uneven compared to even terrains during double support may indicate a greater need for cortical control during this gait phase or as preparation for the following one. The exact timing and involved frequencies of ERDs and event-related synchronizations vary between studies. This may be due to the demands of the walking task, such as treadmill compared to overground walking (99), different gait speeds (67), EEG recording setups (49), and/or analysis methods (67, 100). Future studies may examine whether this effect is specific and reliable and whether it may emerge following left heel strikes with greater sample size or whether an association with leg dominance can be found.

Unfortunately, we only have data on stride times, but not gait speed. Alpha and beta power decreases across the gait cycle at clusters located at central sensorimotor cortices have been observed when increasing gait speeds (40, 67). If altered stride times were associated with altered gait speed, i.e., if the step length remained the same across conditions, the observed condition differences may have been confounded.

No effect of the secondary task on GPMs was discovered, although an effect of the task on stride time was observed. If an effect of task on the GPMs was present but small, the study may not have been sufficiently powered to reveal it, while the size of the behavioral effect and the terrain effect on the GPMs were sufficient. Moreover, we investigated GPMs with a condition-specific baseline, whereas the majority of DTEs on EEG power during walking were observed between conditions [for a review see (33)] and would have been averaged out by our approach. Still, task effects on GPMs were observed while walking and talking compared to walking alone, whereas walking while typing on a smartphone compared to walking alone did not change GPMs (7). Comparing those two dual-task conditions, a greater sustained beta desynchronization during typing compared to talking while walking at electrodes located over the left (pre-)motor cortex was found. This may either suggest that the chosen button-pressing task was not well-suited to reveal effects on GPMs or that the performance of the chosen task may result in sustained, condition-specific changes or changes time-locked to the concurrent task and not GPMs.

This study used a medium number of channels (*N* = 64) and statistical analysis on the sensor level. This may be translated to future (clinical) studies with sparse setups and enables sensor-level analysis of single-subject effects more relevant for clinical application. As shown in Figure 5F, apart from electrodes located over the subjects' neck, likely capturing remaining neck EMG, the greatest gait-related power modulations between 20 and 30 Hz were captured at frontocentral electrodes (around Cz and neighboring electrodes including C1, C2, FC1, FC2, Fz) in all conditions. We hence conclude that by analyzing Cz we managed to investigate a channel capturing much gait-related beta activity. Yet, the greatest power modulation across conditions is not necessarily linked to the differences also being captured at this location. Moreover, this does not necessarily relate to other frequency bands, as previous studies showed that different frequency bands are modulated at different cortical sources during gait (38, 39). These studies have revealed the involvement of clusters localized to various brain regions, like the central sensorimotor, frontal as well as parietal regions (6, 39, 88, 101). Topographies of these clusters suggest that Cz mainly captures the activity of central sensorimotor clusters. In addition, investigating trial and subject averaged data might hide some effects. Artifacts as well as cortical control of gait vary across subjects (38). Previous studies of gait adaptation to external stimuli have revealed neural activity time-locked to these external cues and certain gait events (39). If data on events likely linked to such adaptations would have been available, investigating steps around adaptive events would have been interesting. The goal of this study was to assess the influence of terrain and the performance of a concurrent task on GPMs. Investigating sustained power differences may provide further insight into how networks subserving a certain “status quo” (102) may be affected by concurrent tasks or complex terrain. Finally, as we do not know the neurophysiological effect size, this study might be underpowered.

### Experimental manipulation

#### Terrain difficulty

Following previous findings, participants rated the lawn as more difficult to walk on than the paved footpath but perceived both terrains as rather easy to walk on altogether (103). Previous studies have shown that participants' ratings of difficulty align with increased physical measures of walking complexity (103). Hence, we conclude that the gait difficulty was successfully manipulated with these two terrain choices.

The even and uneven terrain did not only differ in evenness but pavement and lawn also have different dampening characteristics which have been observed to alter gait characteristics (104, 105). As previous studies investigating the effect of uneven terrain altered evenness and surface material at the same time (19–21, 106), these effects cannot be disentangled yet. In the present study, we provide additional evidence that GPMs are a measure sensitive to terrain complexity in natural overground walking, allowing us to study the impact of walking terrain on gait characteristics as well as on cortical signatures of gait systematically.

#### Concurrent task

The button pressing itself is a pure motor task, but since participants were asked to surprise the experimenter with the hand and time of the button press, subjects had to keep track of which and when the last button was pressed. This might have introduced a small cognitive component. To this end, it is similar to the typing of an email that Pizzamiglio et al. (7) investigated. Cognitive tasks requiring internal referencing (e.g., mental tracking) alter gait characteristics to a greater extent than ones relying on external referencing (e.g., reaction time tasks) (8). Internal referencing places a greater demand on networks and/or regions also involved in the control of gait leading to interference effects; tasks involving external referencing activate lower-order networks, interfering less with gait control (107). Choosing a concurrent task that allows performance assessment (e.g., accuracy and reaction times) would have enabled us to investigate both behavioral DTEs, discriminating the effects on gait and task performance (27). Here, we only investigated DTEs of terrain and task on gait parameters, but not on the concurrent task, while complex walking tasks have been linked to performance reduction of a concurrent cognitive task (28, 29).

Moreover, participants were asked to fixate their gaze to a point at eye level, and not to look down at the laptop to maintain a stable gait and successful navigation of the routes, as well as to restrict artifacts introduced by head motion. This avoids confounding the dual-task conditions with altered head motion but impedes the ecological validity as well as the comparability to previous studies (7) since the interaction with electronic devices, such as smartphones, is usually accompanied by looking at their screen. In addition, participants' arm motion was restricted by carrying the recording laptop and pressing the buttons. This may result in a reduced ability to perform recovery movements to maintain balance, for instance following perturbation but has been shown to not impair gait stability in young healthy adults (108). Yet, GPMs may be altered when restricting natural arm swing (44). As arm motions were restricted in all conditions the observed condition difference between even and uneven terrain was likely not influenced by this. Future mobile EEG studies will benefit from smaller hardware featuring higher device mobility (109, 110), thereby enabling the study of brain activity recorded during more natural movement patterns.

### Implications

It is feasible to study GPMs during overground walking outdoors, using mobile EEG in combination with gait detection procedures. Our sensor-level analysis yielded comparable results to previous analyses in source space and could replicate GPMs in a more ecologically valid setup. For this dataset, a previously proposed method for reducing muscle artifacts (57) outperformed other preprocessing strategies. GPMs could not only be compared across different terrain but also with and without the performance of a concurrent task. This highlights that more than general power differences between conditions or neural correlates of a concurrent task can be investigated. Yet, different GPMs were only observed with altered terrain but not with concurrent task performance. Hence, in this study, we provide evidence that terrain complexity alters gait control demands, and that the associated cortical processes can be identified with mobile EEG.

## Data availability statement

The datasets presented in this study can be found in online repositories. The names of the repository/repositories and accession number(s) can be found below: https://openneuro.org/datasets/ds003039/versions/1.0.2. MATLAB code is available at GitHub (https://github.com/NadineJac/gaitEEG_task_terrain/releases/tag/v1.0.1). Experimental files can be accessed via OSF (doi:10.17605/OSF.IO/A8QCM).

## Ethics statement

The studies involving human participants were reviewed and approved by the Medizinische Ethikkommission der Carl von Ossietzky Universität Oldenburg, University of Oldenburg, Oldenburg, Germany. The patients/participants provided their written informed consent to participate in this study.

## Author contributions

NJ collected the data and wrote the analysis scripts. SD supervised the project. All authors contributed to the design of the study, writing the manuscript and approved the submitted version.

## Funding

This project was supported by intramural funds from the University of Oldenburg Faculty IV School of Medicine and Health Sciences (Forschungspool), and the BMBF Project NeuroCommTrainer 16SV7790.

## Conflict of interest

Author SB was employed by the company Hörzentrum Oldenburg GmbH. The remaining authors declare that the research was conducted in the absence of any commercial or financial relationships that could be construed as a potential conflict of interest.

## Publisher's note

All claims expressed in this article are solely those of the authors and do not necessarily represent those of their affiliated organizations, or those of the publisher, the editors and the reviewers. Any product that may be evaluated in this article, or claim that may be made by its manufacturer, is not guaranteed or endorsed by the publisher.

## Footnotes

^1^ https://github.com/NadineJac/gaitEEGfootprint/releases/tag/v1.2
