# Supplementary material for: Mobile electroencephalography captures differences of walking over even and uneven terrain but not of single and dual-task gait
Source: Front Sports Act Living. 2022 Oct 6;4:945341. doi: 10.3389/fspor.2022.945341 (PMC9582531; doi:10.3389/fspor.2022.945341)
Supplement: Supplementary file 1 [file Data_Sheet_1.PDF]

Supplementary Material

Several post independent component analysis (ICA) artifact attenuation strategies were compared to choose the one with the best sensitivity for this dataset. All strategies have similarities to previously used artifact attenuation approaches in gait EEG or recommended settings of the employed tools. These strategies encompass rejection of different independent components (ICs)(pipelines A and B), clustering of ICs with repetitive clustering (pipelines C and D), and spectral PCA (pipeline E).

1 Methods

The five post ICA artifact attenuation strategies described below were compared regarding the reduction of gait-related artifacts and remaining patterns of gait-related activity compared to unprocessed data. As depicted in Supplementary Figure S1 and described in section 2.4 *Independent Component Analysis* of the main article, data were prepared, and ICA weights were obtained before the artifact attenuation diverged.

1.1 Pipeline A: Keep brain ICs

In line with previous studies (Scanlon, Jacobsen, Maack, & Debener, 2020, 2022; Stuart, Wagner, Makeig, & Mancini, 2021), only ICs that were labeled by IClabel (v1.3) (Pion-Tonachini, Kreutz-Delgado, & Makeig, 2019) as brain with at least 70% probability were kept.

1.2 Pipeline B: Reject artifactual ICs

ICs that were labeled as eye and muscle with at least 90% probability were rejected. These are the default rejection settings of IClabel (v1.3) (Pion-Tonachini et al., 2019).

1.3 Pipeline C: Repetitive clustering of all ICs

Clustering of equivalent dipole locations is commonly applied in gait EEG studies (Gwin, Gramann, Makeig, & Ferris, 2011; Malcolm et al., 2020; Nordin, Hairston, & Ferris, 2020; Sipp, Gwin, Makeig, & Ferris, 2013). In order to improve the reliability of obtained solutions we used a repetitive clustering approach (Gramann, Hohlefeld, Gehrke, & Klug, 2021) as implemented in the BeMoBiL pipeline (Klug, Gehrke, Hohlefeld, & Gramann, 2018) (available at https://github.com/BeMoBIL/bemobil-pipeline).

Before clustering, dipoles were fitted to all ICs using DIPFIT (version 4.1)(Oostenveld, Delorme, & Makeig, 2003) with a three-layer boundary element model with the respective standard magnet resonance imaging and channel locations. Data were epoched from 0 to 1.2 s around right heel strikes. The mean amplitude of each epoch was subtracted as baseline correction. Epochs in which the order of gait evens did not follow a usual gait cycle (right heel-strike, left toe-off, left heel-strike, right toe-off, right heel-strike) were excluded. Furthermore, epochs with a joint probability greater than 3 SDs were rejected. IC event-related potentials (ERPs), scalp topographies, and spectra were calculated and a weighted (ERP: 1, topography: 1, spectra: 1, equivalent dipole location: 3) pre-clustering array from 3 to 25 Hz across the whole epoch was constructed.

The data were clustered into n = 50 clusters 1000 times with a k-means algorithm. ICs were considered outliers if they were further than three standard deviations apart from any cluster centroid. Clusters were optimized to a region of interest (ROI). An ROI obtained with functional magnetic resonance imaging in the primary sensorimotor cortex (SMC) active during foot motions (Talairach coordinates: x = -1, y = -26, z = 62) was chosen (Sahyoun, Floyer-Lea, Johansen-Berg, & Matthews, 2004). The obtained solutions were ranked according to a weighted sum of the standardized quality measures, and the best fitting cluster was chosen. Cluster quality was assessed as:

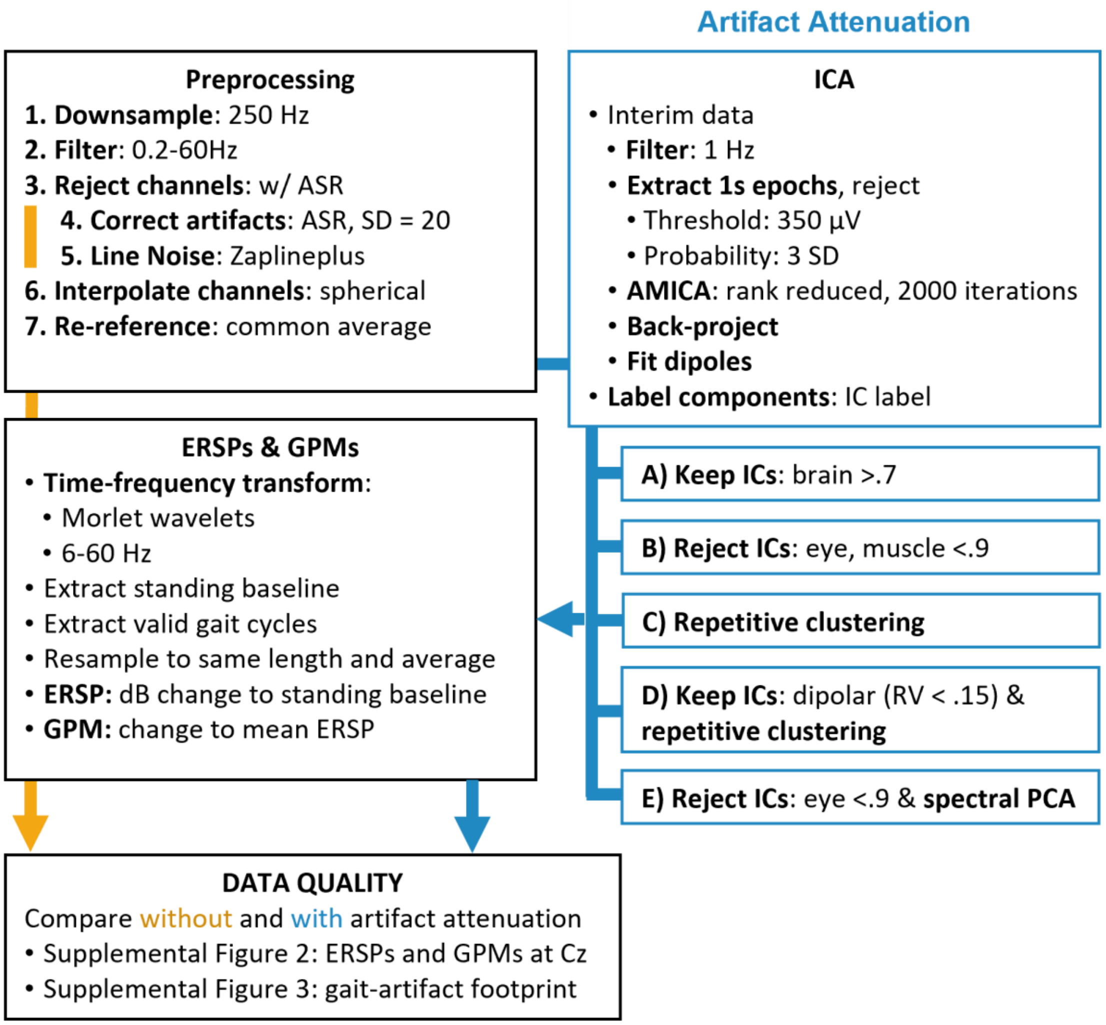

Supplementary Figure 1. Outline of EEG preprocessing and comparison of different post-ICA artifact attenuation approaches.

(1) the number of participants with at least one IC in the cluster (weight: 3),
(2) the ratio of the number of ICs to the number of participants (weight: -1),
(3) the normalized cluster spread (weight: -1),
(4) the mean residual variance (RV) of the fitted dipoles (weight: -1)
(5) distance of the cluster centroid to the ROI (weight: -2)
(6) Mahalanobis distance to the median of the multivariate distribution of the obtained quality measures (1-5) of all solutions (weight: -1)

ICs of the best fitting cluster were projected to the electrode locations and mean gait cycle ERSPs and GPMs were calculated for each subject as described in section 1.6.

1.4 Pipeline D: Repetitive clustering of dipolar ICs

Repetitive clustering was performed and the best fitting cluster was chosen as described in section 1.3, but only the ICs with a residual variance of less than 15% and an equivalent dipole location inside the head were clustered (Gwin et al., 2011; Malcolm et al., 2020; Nordin et al., 2020; Sipp et al., 2013).

1.5 Pipeline E: spectral principal component analysis

Spectral principal component analysis (PCA)(Seeber, Scherer, Wagner, Solis-Escalante, & Müller-Putz, 2015) was performed as described in the manuscript in section 2.4 *spectral principal component analysis*.

1.6 Evaluation of artifact attenuation

Following artifact attenuation, data not already time-frequency-transformed were transformed, resampled to the same unit length, and averaged over gait cycles, as described in section 2.4 *independent component analysis*. Gait artifacts were compared using the modified footprint with the following criteria: (B) explained variance across frequencies, (C) power ratio of lateral to medial channels, (D) power ratio at electrodes located over the neck following contralateral compared to ipsilateral heal strikes, (E) ratio during double compared to single support phases. A detailed description can be found in the main article in section 2.5 *EEG data quality*. The code is available at https://github.com/NadineJac/gaitEEGfootprint. As two of the artifact attenuation strategies employed clustering of ICs which aggregates data over subjects, data were only compared at group-level. Descriptively it was assessed whether the artifact extent decreased with artifact attenuation, as reflected by smaller footprint feature values. The gait-related activity was evaluated at Cz, the electrode also chosen for the analysis in the main article. We looked for alpha/mu and beta power desynchronizations compared to a standing baseline (event-related spectral perturbations, ERSPs) and for gait-related power modulations (GPM) compared to a mean gait cycle baseline. Qualitatively, we assumed alpha and beta power decreases compared to a standing baseline (ERSP) and power modulations over the gait cycle like beta power decreases during double support and increases during single support (GPM) to be neural correlates of gait and thus indicative of successful artifact attenuation. Equivalent dipole locations, cluster scalp topographies, and cluster ERSPs and GPMs of the involved ICs (not their projection to Cz) of the best fitting clusters (pipeline C and D) are visualized.

Custom MATLAB scripts are available at https://github.com/NadineJac/gaitEEG_postICAopt.

2 Results

2.1 Pipeline A: Keep brain ICs

On average 13.89 ICs (range 9 - 22 ICs) per participant were classified as brain with at least 70% probability and kept.

2.2 Pipeline B: Reject artifactual ICs

On average 2.63 ICs (range 2 – 4 ICs) per participant were classified as eye and 6.74 (range 1 – 16 ICs) as muscle and rejected.

2.3 Pipeline C: Repetitive clustering

On average 60.63 ICs (range 58 – 63 ICs) per participant were submitted to the repetitive clustering. The best-fitting cluster contained 23 ICs from 15 subjects, so on average 1.53 ICs per participant. The mean RV was 30.7%, the cluster spread was 370.2, and the distance to the ROI was 14.2 Talairachian coordinate units (see Supplementary Figure 2a).

The cluster topography displayed the most activity around the vertex (see Supplementary Figure 2c). The cluster ERSP showed a strong power decrease around 50 Hz and a power increase at frequencies below 6 Hz. Power increased during double support (approximately 0 to 16% and 50 to 66% of the gait cycle) from around 10 to 14 Hz, 38 to 46, and 54 to 60 Hz (see Supplementary Figure 2e). The cluster GPM displayed broadband power increases during double support (approximately 0 to 16% and 50 to 66% of the gait cycle), strongest between approximately 8 to 24 Hz (see Supplementary Figure 2g).

2.4 Pipeline D: Repetitive clustering of dipolar ICs

On average 23.00 ICs (range 9 - 33 ICs) were kept per participant and submitted to the repetitive clustering. The best-fitting cluster contained 14 ICs from 12 subjects, so on average 1.17 ICs per participant. The mean RV was 7.1%, the cluster spread was 218.3, and the distance to the ROI was 19.42 Talairachian coordinate units (see Supplementary Figure 2b).

The cluster topography displayed the most activity around the vertex, slightly more posterior than cluster C (see Supplementary Figure 2d). The cluster ERSP was dominated by desynchronizations from 4 to 60 Hz over the whole gait cycle and dominated by a strong power decrease around 50 Hz (see Supplementary Figure 2f). The cluster GPM displayed small, broadband power increases during double support (approximately 0 to 16% and 50 to 66% of the gait cycle), strongest between approximately 40 to 60 Hz. During single support, small beta power increases from around 24 to 32 Hz and 25 to 50%, and 75% to 95% of the gait cycle emerged (see Supplementary Figure 2h).

2.5 Pipeline E: spectral cleaning

On average 2.63 ICs (range 2 – 4 ICs) per participant were identified as representing eye artifacts and rejected.

2.6 Evaluation of artifact attenuation

Descriptively, some features of the gait artifact footprint were altered by artifact attenuation while others were not. Feature B, the explained variance across frequencies remained largely unchanged by artifact processing but was greatly reduced with pipeline E (see Supplementary Figure 3 and Supplementary Table 1). Feature C, the ratio of power at lateral compared to medial channels was descriptively marginally reduced by pipelines C, D, and B, and reduced to a greater extent by pipelines A and E. Feature D, the power ratio at electrodes located over the neck following contralateral compared to ipsilateral heel-strikes did descriptively increase slightly following pipeline B, C, and D, but decrease to a similar extend following pipeline A and E. Feature F, the standing/walking power ratio, was reduced following all artifact attenuation approaches, in descending order the decrease was greatest following pipeline E, A, D, B, and C. In summary, the gait artifact footprint decreased the most with pipeline E followed by pipeline A.

ERSPs displayed alpha power (here 8 to 12 Hz) decreases during single support (approximately 16 to 50% and 66 to 100% of the gait cycle) without and with artifact processing (see Supplementary Figure 4). Beta power (here 14 to 28 Hz) decreased compared to a standing baseline. These decreases were interrupted broadband power increases following heal strikes (0% and 50% of the gait cycle) without artifact attenuation. This pattern was strengthened with pipelines C and D, but diminished with pipelines A, B and is absent with pipeline E. GPMs showed greater broadband activity following heel strikes (0% and 50% of the gait cycle) without artifact attenuation and with pipeline B, C and D. The extent of the broadband power increases was diminished with pipeline A and some beta power increases emerged following the left toe-off (approximately 16% of the gait cycle). With pipeline E, beta power decreased during double support (approximately 0 to 16% and 50 to 66% of the gait cycle) and increased during single support.

3 Decision

The gait-related artifact footprint was reduced the most by pipeline E. This pipeline moreover altered the putatively artifactual broadband GPM activity following heal strikes, dominant without artifact attenuation, to the greatest extent. This revealed patterns similar to previous studies of gait-related neural activity (Seeber, Scherer, Wagner, Solis-Escalante, & Müller-Putz, 2014; Seeber et al., 2015; Wagner et al., 2012). Hence, we decided to use pipeline E for the main analysis as reported in the manuscript. Note that cluster GPMs following the repetitive clustering of dipolar ICs (pipeline D) also displayed some beta power increases during single support, which we assume to be linked to gait-related neural activity. This pattern is not visible at channel Cz, possibly emphasizing that the benefit of clustering ICs is greatest when analyzing the obtained cluster instead of back-projecting the ICs to the sensor level.

3.1.1 Limitation

Artifact attenuation does not only need to be sensitive but also specific to the artifacts it is supposed to attenuate and not reduce the neural activity of interest. Yet, we refrained from assessing the specificity of the artifact attenuation by evaluating another, non-gait-locked ERP, i. e. the auditory evoked potential (e. g. Hine & Debener, 2007) of the feedback sound of the button-pressing (cf. Jacobsen, Blum, Witt, & Debener, 2020). This was necessary as suitable data were not available after all processing pipelines. For instance, pipeline E produced gait ERSPs and GPMs, so that the calculation of a non-gait locked ERP was not possible anymore. Moreover,

clusters obtained by pipelines C and D coul be used to calculate an auditory evoked potential, but tha data may not underestimate ist, as ICs were clustered based on similarities of EEG features time-locked to the gait cycle around an ROI associated with motor control and not auditory processing.

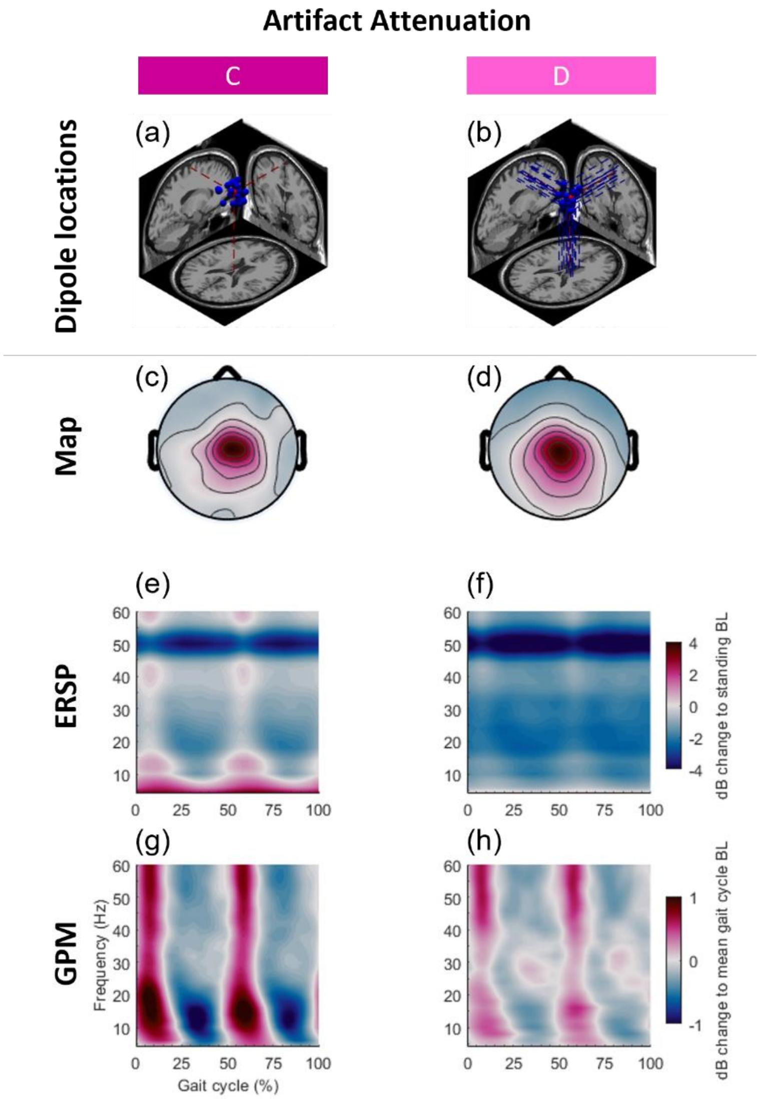

Supplementary Figure 2. Details on best fitting clusters following artifact attenuation approach C (left column) and D (right column). Dipole locations (A, B), cluster topographies (C, D), ERSPs at Cz (E, F), and gait-phase related power modulations (G, H) for both clusters respectively

Supplementary Table 2. Footprint features B to F of group averaged data without artifact attenuation or with pipelines A to E

**pipeline**	**feature**				
	B	C	D	E	F
raw	0.97	1.96	1.60	1.01	1.82
Pipeline A	0.93	1.38	1.17	1.00	0.82
Pipeline B	0.97	1.70	1.86	1.42	1.22
Pipeline C	0.97	1.94	1.75	1.02	1.37
Pipeline D	0.96	1.79	1.80	1.09	1.13
Pipeline E	0.00	0.82	1.24	0.84	0.16

Note. Footprint features B to E of group averaged unprocessed data and following processing pipelines A to E

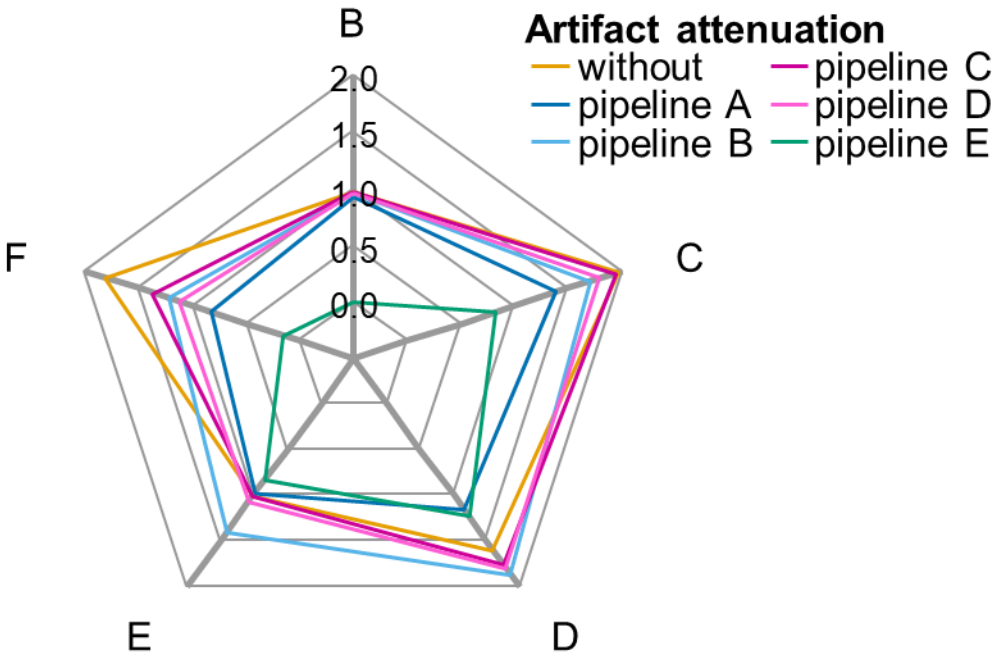

Supplementary Figure 3. Gait Artifact related footprint of EEG data without and with different post-ICA artifact attenuation strategies. Features are (B) Explained variance across frequencies, (C) power ratio of lateral to medial channels, (D) power ratio at electrodes located over the neck following contralateral compared to ipsilateral heal strikes, (E) ratio during double compared to single support, (F) standing/walking power ratio, as described in Jacobsen et al. (2020).

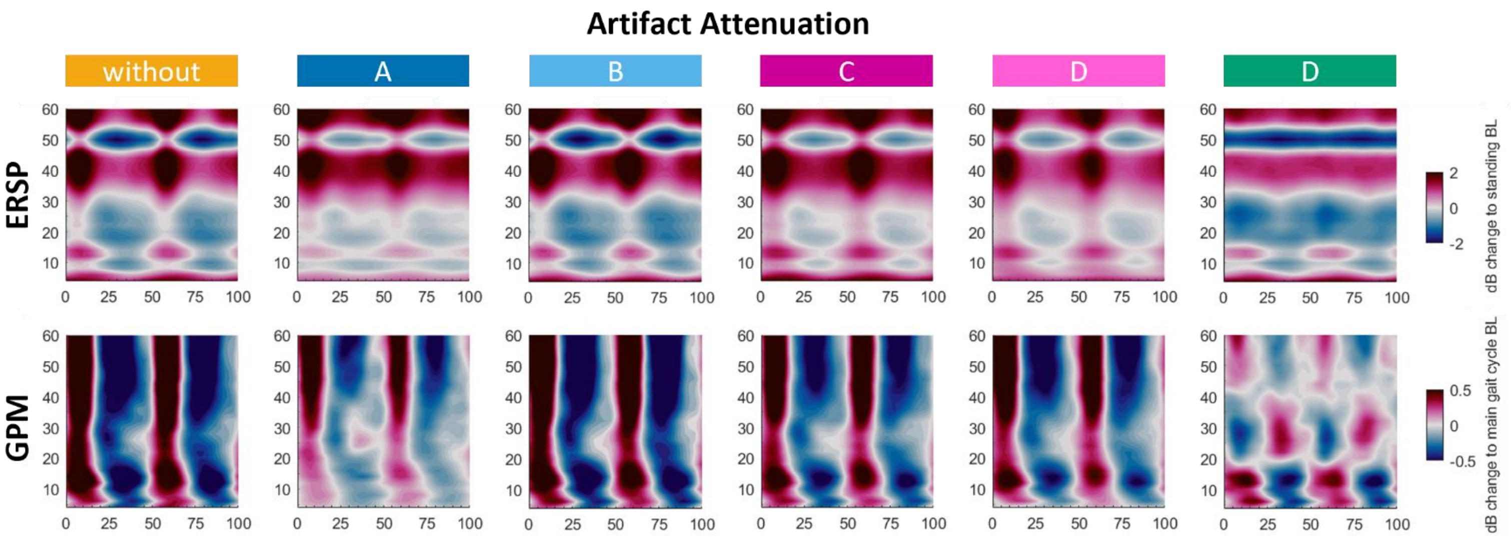

Supplementary Figure 4. Event-related spectral perturbations (ERSPs, top row) and gait-phase related power modulations (GPM, bottom row) without artifact attenuation (right column) and with artifact attenuation pipeline A to E (from left to right)

4 References

Gramann, K., Hohlefeld, F. U., Gehrke, L., & Klug, M. (2021). Human cortical dynamics during full-body heading changes. *Scientific Reports*, *11*(1), 18186. https://doi.org/10.1038/s41598-021-97749-8

Gwin, J. T., Gramann, K., Makeig, S., & Ferris, D. P. (2011). Electrocortical activity is coupled to gait cycle phase during treadmill walking. *NeuroImage*, *54*(2), 1289–1296. https://doi.org/10.1016/j.neuroimage.2010.08.066

Hine, J., & Debener, S. (2007). Late auditory evoked potentials asymmetry revisited. *Clinical Neurophysiology*, *118*(6), 1274–1285. https://doi.org/10.1016/j.clinph.2007.03.012

Jacobsen, N. S. J., Blum, S., Witt, K., & Debener, S. (2020). A walk in the park? Characterizing gait-related artifacts in mobile EEG recordings. *European Journal of Neuroscience*. https://doi.org/10.1111/ejn.14965

Klug, M., Gehrke, L., Hohlefeld, F. U., & Gramann, K. (2018). The BeMoBIL Pipeline – Facilitating Mobile Brain / Body Imaging (MoBI) Data Analysis in MATLAB, (July), 2–3.

Malcolm, B. R., Foxe, J. J., Joshi, S., Verghese, J., Mahoney, J. R., Molholm, S., & De Sanctis, P. (2020). Aging-related changes in cortical mechanisms supporting postural control during base of support and optic flow manipulations. *European Journal of Neuroscience*, (October). https://doi.org/10.1111/ejn.15004

Nordin, A. D., Hairston, W. D., & Ferris, D. P. (2020). Faster Gait Speeds Reduce Alpha and Beta EEG Spectral Power From Human Sensorimotor Cortex. *IEEE Transactions on Biomedical Engineering*, *67*(3), 842–853. https://doi.org/10.1109/TBME.2019.2921766

Oostenveld, R., Delorme, A., & Makeig, S. (2003). DIPFIT: Equivalent dipole source localization of independent components. Retrieved from https://github.com/sccn/dipfit

Pion-Tonachini, L., Kreutz-Delgado, K., & Makeig, S. (2019). The ICLabel dataset of electroencephalographic (EEG) independent component (IC) features. *Data in Brief*, *25*, 104101. https://doi.org/10.1016/j.dib.2019.104101

Sahyoun, C., Floyer-Lea, A., Johansen-Berg, H., & Matthews, P. M. (2004). Towards an understanding of gait control: Brain activation during the anticipation, preparation and execution of foot movements. *NeuroImage*, *21*(2), 568–575. https://doi.org/10.1016/j.neuroimage.2003.09.065

Scanlon, J. E. M., Jacobsen, N. S. J., Maack, M. C., & Debener, S. (2020). Does the electrode amplification style matter? A comparison of active and passive EEG system configurations during standing and walking. *European Journal of Neuroscience*, *44*(8), ejn.15037. https://doi.org/10.1111/ejn.15037

Scanlon, J. E. M., Jacobsen, N. S. J., Maack, M. C., & Debener, S. (2022). Stepping in time: Alpha-mu and beta oscillations during a walking synchronization task. *NeuroImage*, *253*(December 2021), 119099. https://doi.org/10.1016/j.neuroimage.2022.119099

Seeber, M., Scherer, R., Wagner, J., Solis-Escalante, T., & Müller-Putz, G. R. (2014). EEG beta suppression and low gamma modulation are different elements of human upright walking. *Frontiers in Human Neuroscience*, *8*, 485. https://doi.org/10.3389/fnhum.2014.00485

Seeber, M., Scherer, R., Wagner, J., Solis-Escalante, T., & Müller-Putz, G. R. (2015). High and low gamma EEG oscillations in central sensorimotor areas are conversely modulated during the human gait cycle. *NeuroImage*, *112*, 318–326. https://doi.org/10.1016/j.neuroimage.2015.03.045

Sipp, A. R., Gwin, J. T., Makeig, S., & Ferris, D. P. (2013). Loss of balance during balance beam walking elicits a multifocal theta band electrocortical response. *Journal of Neurophysiology*, *110*(9), 2050–2060. https://doi.org/10.1152/jn.00744.2012

Stuart, S., Wagner, J., Makeig, S., & Mancini, M. (2021). Brain Activity Response to Visual Cues for Gait Impairment in Parkinson's Disease: An EEG Study. *Neurorehabilitation and Neural Repair*, *35*(11), 996–1009. https://doi.org/10.1177/15459683211041317

Wagner, J., Solis-Escalante, T., Grieshofer, P., Neuper, C., Müller-Putz, G., & Scherer, R. (2012). Level of participation in robotic-assisted treadmill walking modulates midline sensorimotor EEG rhythms in able-bodied subjects. *NeuroImage*, *63*(3), 1203–1211. https://doi.org/10.1016/j.neuroimage.2012.08.019
